# Role of OCT-4 and NANOG in the Malignant Transformation of Oral Submucous Fibrosis: An Immunohistochemical Evaluation

**DOI:** 10.7759/cureus.96351

**Published:** 2025-11-07

**Authors:** Diksha Mohapatra

**Affiliations:** 1 Department of Oral Pathology and Microbiology, Institute of Dental Sciences, Siksha 'O' Anusandhan (Deemed to be University), Bhubaneswar, IND

**Keywords:** cancer stem cell markers, diagnosis, epithelial-mesenchymal transition (emt), immunohistochemistry (ihc), oral cancers, oral squamous cell carcinoma, oral submucous fibrosis

## Abstract

Background: Oral squamous cell carcinoma (OSCC) is one of the most common cancers of the oral cavity detected worldwide due to delayed clinical diagnosis, expensive therapeutic procedures, and poor prognosis. Oral submucous fibrosis (OSMF) is an oral potentially malignant disorder (OPMD) marked by abnormal collagen production which has a high malignant transformation rate. Subsequent changes occur in the epithelium and connective tissue simultaneously in a coordinated manner and can be aptly mentioned as epithelial-mesenchymal transition (EMT) that facilitates invasiveness and metastasis in carcinomas. The study was conducted to evaluate the immunohistochemical expression of cancer stem cell markers (CSM) OCT-4 and NANOG in OSMF (OSMF) patients, OSCC transformed from OSMF (OSCC+OSMF) patients, and OSCC not transformed from OSMF (OSCC-OSMF) patients. Additionally, the study sought to correlate the immunoexpression levels of OCT-4 and NANOG with the demographic and clinicopathological characteristics of OSMF, OSCC+OSMF, and OSCC-OSMF patients.

Methodology: A total of 86 patients (30 OSMF, 30 OSCC+OSMF, and 26 OSCC-OSMF) were included in the study, wherein 86 haematoxylin and eosin-stained sections, 86 immunohistochemistry slides with OCT-4, and 86 immunohistochemistry slides with NANOG of the same patients were examined to analyse the role of OCT-4 and NANOG immunoexpression in the malignant transformation of OSMF.

Result: A high proportion of OSMF (96.6%) and OSCC+OSMF (93.3%) samples showed positive OCT-4 expression, while only 42.3% of OSCC-OSMF cases were positive (p<0.001). NANOG showed a similar trend, being expressed in 93.3% of OSMF, 83.3% of OSCC+OSMF, and 38.5% of OSCC-OSMF samples (p<0.001). Both markers exhibited stronger staining intensity and higher immunoreactive scores in OSMF and OSCC+OSMF than in OSCC-OSMF. Odds ratio (OR) analysis indicated that OSCC+OSMF cases had significantly higher odds of OCT-4 (OR=5.23) and NANOG (OR=12.5) expression compared to OSCC-OSMF.

Conclusion: The elevated expression of OCT-4 and NANOG in OSMF and OSCC+OSMF suggests their involvement in stem cell-driven pathways underlying malignant transformation. These markers may serve as valuable indicators for identifying OSMF patients at higher risk of developing OSCC and hold potential as prognostic and therapeutic indicators in oral carcinogenesis.

## Introduction

Oral submucous fibrosis (OSMF) is an oral potentially malignant disorder (OPMD) marked by abnormal collagen production having a 6% malignant transformation rate ranging from 1.2% to 23% into oral squamous cell carcinoma (OSCC) [1]. According to a statistical survey by the World Health Organization, more than five million cases of OSMF have been reported globally [2]. India has the highest number of OSMF cases with an areca nut consumption rate of 23.9%, though the condition is also prevalent in other Asian regions, where there is widespread consumption of areca nut and betel quid which has a strong etiological association in the pathogenesis of OSMF [3]. According to survey, epidemiologic, and clinical studies, 7.6% of patients diagnosed with OSMF develop oral cancers in India within 3-16 years of detection [4]. OSCC is one of the 10 most common cancers detected worldwide and accounts for nearly 80-90% of all malignancies of the oral cavity, with delayed clinical diagnosis, expensive therapeutic procedures, and poor prognosis. The overall risk of developing oral cancer is one in 60 (1.7%) in men and one in 140 (0.71%) in women with a 0.4% per year increase in mortality rate as per a statistical survey conducted by the American Cancer Society. According to a survey on cancer mortality in India, 71% of cancer deaths occurred in people aged between 30 and 69 years, out of which tobacco-related cancer death was seen in 42% of male patients and 18.3% of female patients [5].

The characteristics of OSMF are excessive collagen accumulation in the submucosa, resulting from collagen distribution disorder as a result of areca nut alkaloid exposure. There is an occurrence of noticeable histopathological feature changes in the stroma such as reduced vascularity, reduced density of inflammatory cells, and sub-epithelial hyalinisation that may influence the process of malignant transformation. Inadequate oxygen supply and hypoxia upregulate hypoxia-inducible factor 1-alpha (HIF-1α), inducible nitric oxide synthase (INOS), platelet-derived growth factor (PDGF), and fibroblast growth factor (FGF) expression which is responsible for the activation of pro-angiogenic genes that encodes vascular endothelial growth factor (VEGF) and transforming growth factor-beta (TGF-β) expression, causing increased angiogenesis, cell proliferation, and cell survival [6,7]. Arecoline in areca nut causes the nitrosation of areca alkaloids and induces the release of interleukins 6 and 8 and GRO-α causing oxidative damage to the tissue and releasing reactive oxygen species (ROS) which leads to genetic damages by breaking the double-stranded DNA strands in the keratinocytes [8]. Arecoline also alters the expression of various onco-suppressor genes and anti-apoptotic molecules, thus aiding in the malignant transformation of OSMF [9].

Subsequent changes occurring in the epithelium and connective tissue simultaneously in a coordinated manner can be aptly mentioned as epithelial-mesenchymal transition (EMT) that facilitates invasiveness and metastasis in carcinomas [10]. In the multi-step process of tumorigenesis, EMT, a latent embryonic developmental program, is reactivated, prompting the initiation of molecular and cellular pathways that promote genetic mutations. This enables cancer cells to acquire replicative properties, growth factor independence, resistance to growth-inhibitory signals, as well as enhanced capacity for tissue invasion and metastasis. A tumour in bulk consists of rapidly multiplying cells known as transit-amplifying cells and post-mitotic differentiated cells which do not participate in tumour initiation. These cells source their origin from cancer stem cells (CSCs) through differentiation, but themselves do not cause tumour initiation. The CSC model hypothesis states that mutation can convert normal somatic cells into cancerous or transform cancer cells into stem cell-like which undergo self-renewal, proliferation, differentiation, and adaptation and control the immune system, thus mediating tumorigenesis, metastasis, and resistance to therapy [11]. Cells undergoing EMT can acquire stem-cell-like properties where epithelial cells with induced EMT can show properties similar to mesenchymal cells like genetic expression, multidirectional differentiation, and migration towards wound sites [12].

Till date, very few studies have been conducted on CSC markers to understand their role in the malignant transformation of OSMF to OSCC. Therefore, we have hypothesised that the expression of OCT-4 and NANOG, two CSC markers, may play an important role in the malignant transformation of OSMF and conducted the present study to evaluate the immunohistochemical expression of OCT-4 and NANOG in OSMF (OSMF) patients, OSCC transformed from OSMF (OSCC+OSMF) patients, and OSCC not transformed from OSMF (OSCC-OSMF) patients and to associate the expression of OCT-4 and NANOG with the demographic and clinicopathological features of OSMF, OSCC+OSMF, and OSCC-OSMF.

## Materials and methods

The study was reviewed independently and approved by the Institutional Ethics Committee of the Institute of Medical Sciences (IMS) and SUM Hospital, Siksha 'O' Anusandhan (Deemed to be University), Bhubaneswar, Odisha, India (approval number: ECR/627/Inst/OR/2014/RR-20). Before sample collection, the participants/legal guardians were informed and explained in detail about the study, and written consents were obtained before proceeding with the study. In case of deceased patients, informed consents were obtained from their respective legal guardians. The study was performed in accordance with the Declaration of Helsinki [13].

Study design

In this prospective and retrospective case-control study, a sample size of 86 (30 OSMF, 30 OSCC+OSMF, 26 OSCC-OSMF) has been analysed for demographic data, clinical features, histopathological diagnostic features in haematoxylin and eosin staining, and immunohistochemistry (IHC) with OCT-4 and NANOG. The demographic data such as age, sex, habit history, and duration of habit were collected from the three groups. The TFM (Trismus, Fibrosis, Malignant Transformation) Classification was used to clinically stage OSMF patients by assessing the degree of trismus, extent of fibrosis, and presence of malignant transformation. The clinical features such as mouth opening (in mm), clinical stage (according to mouth opening), tongue protrusion, burning sensation, presence of fibrotic bands, presence of white patch and blanching, palatal movement, and uvula appearance were recorded in groups 1 and 2. The TNM (Tumour, Node, Metastasis) staging was an additional clinical feature in groups 2 and 3. The histopathological features in haematoxylin and eosin-stained sections of group 1 were epithelial thickness, juxtaepithelial hyalinisation, juxtaepithelial inflammatory cell infiltration, and degree of dysplasia. Histopathological grading was an additional feature recorded in groups 2 and 3. Immunohistochemical evaluation was done, wherein the qualitative parameter was the intensity of staining of immunohistochemical markers and the quantitative parameter was the percentage of immunohistochemically stained positive cell population. By combining both qualitative and quantitative values, the immunoreactive score (IRS) was calculated [14].

Tissue and cellular localisation of markers were the additional immunohistochemical parameters recorded.

Study setting

Formalin-fixed paraffin-embedded (FFPE) incisional biopsy specimens of the included cases were collected prospectively from August 20, 2020, to December 31, 2022, and retrospectively from August 20, 2020, to December 1, 2014, from the patient records of the Department of Oral Pathology, Institute of Dental Sciences, Siksha 'O' Anusandhan (Deemed to be University), Odisha, India.

Study participants

The FFPE tissues of OSMF (group 1; n=30), OSCC+OSMF (group 2; n=30), and OSCC-OSMF (group 3; n=26) were retrieved along with their demographic, clinical, and histopathological features. Specimens from the three groups were classified based on tumour size, lymph node involvement, and metastatic status assessed during clinical examination and subsequently processed for sectioning and staining using routine haematoxylin and eosin along with immunohistochemical markers OCT-4 and NANOG. Clinically and histopathologically diagnosed OSMF, OSCC+OSMF, and OSCC-OSMF cases were included in this study. Cases with biopsy specimens from carcinomas originating in sites other than the oral cavity (like oropharyngeal carcinoma, oesophageal carcinoma, and nasopharyngeal carcinoma) or metastatic or recurrent carcinomas and those cases without documented patient consent were excluded from the study. The details of immunohistochemical markers have been given in Table 1.

**Table 1:** Details of immunohistochemical markers used IgG: immunoglobulin G; EDTA: ethylenediaminetetraacetic acid; KLH: keyhole limpet hemocyanin; PBS: phosphate-buffered saline; BSA: bovine serum albumin

Markers	Primary antibody	Secondary antibody	Dilution	Storage	Clone	Immunogen	Antigen retrieval solution	Buffer
OCT-4	Rabbit monoclonal antibody	IgG	1.50-1.100	2-8°C	EP143	A synthetic peptide corresponding to residues of the human OCT-4 protein	Tris-EDTA	pH 9.7
NANOG	NANOG polyclonal antibody	IgG	1.300-1.800	-20°C	Q80Z64	KLH conjugated synthetic peptide corresponding to mouse NANOG	PBS with 0.02% sodium azide, 1% BSA, and 50% glycerol	pH 7.4

Immunohistochemical study

Immunohistochemical analysis for OCT-4 and NANOG expression was done in 86 FFPE specimens (30 OSMF, 30 OSCC+OSMF, 26 OSCC-OSMF) using the standard immunoperoxidase technique.

Evaluation of haematoxylin and eosin-stained slides

Epithelial thickness, presence and absence of juxtaepithelial hyalinisation, presence and absence of juxtaepithelial inflammation, and degree of dysplasia according to the WHO grading system for oral epithelial dysplasia [15] were evaluated in the haematoxylin and eosin-stained slides of group 1. Tumour grading according to the WHO grading system [16] was observed in groups 2 and 3.

Evaluation of IHC slides

The qualitative expression of OCT-4 and NANOG was evaluated by observing staining intensity. The scoring criteria used were as follows: 0 (no staining), 1 (weak staining), 2 (moderate staining), and 3 (strong staining) [17]. Quantitative expression was evaluated by the proportion of positively stained cells and reported as four grades: 0 (no positively stained cells), 1 (<10% positively stained cells), 2 (10-50% positively stained cells), and 3 (>50% positively stained cells) [17].

The IRS was calculated by multiplying the percentage score of positive cells with the score of staining intensity, the details of which are shown in Table 2. The IRS was computed by multiplying areas of tumour staining by the staining intensity score (0, 1, 2, 3, 4, 6, 9). Tumours with a staining score of ≤4 and ≥6 were considered to have low and high expression, respectively, as per this technique of evaluation. A light microscope was used for the evaluation of all the haematoxylin and eosin-stained sections as well as IHC-stained sections at 400× magnification [17]. 

**Table 2:** IRS scoring system Percentage of positive cells×intensity of staining=IRS (0-9), where IRS ≤4=low expression and IRS ≥6=high expression. Modified based on the scoring criteria described in [17] for clarity and reader understanding. The design, structure, and presentation of the table are original, created specifically for this manuscript and not reproduced from any previously published source. IRS: immunoreactive score

Scoring criteria used for the proportion of positively stained cells	Scoring criteria used for staining intensity
No positive tumour cells: 0	No staining: 0
<10% positive tumour cells: 1	Weak staining: 1
10-50% positive tumour cells: 2	Moderate staining: 2
>50% positive tumour cells: 3	Strong staining: 3

Mouth opening in OSMF and OSCC+OSMF, clinical staging (according to mouth opening) in OSMF, epithelial thickness in OSMF, degree of dysplasia in OSMF, tumour grading in OSCC+OSMF and OSCC-OSMF, and TNM staging in OSCC+OSMF and OSCC-OSMF were reported by the same examiner to avoid any bias caused due to subjective interpretation.

Study size

Sample size calculation was done after validating the data internally. Sample size was estimated in the G*Power software Ver 3.1.9.7 (Heinrich-Heine-Universität Düsseldorf, Düsseldorf, Germany) estimation based on the specified effect size, power (typically 80-90%), and significance level (alpha) which resulted in 30 samples in each group with high precision (5% type I error) [18].

Statistical method

In the study, the SPSS software version 17.0, developed by SPSS Inc., Chicago, IL, USA, was used for conducting statistical analysis. Different groups were compared using Fisher's exact test, and a p-value of less than 0.05 was considered to be statistically significant. Odds ratios of OCT-4 and NANOG immunoexpression were assessed using the jamovi 2.3.28 software (jamovi [Computer Software]. Retrieved from https://www.jamovi.org). Mouth opening in OSMF, clinical staging (according to mouth opening) in OSMF, mouth opening in OSCC+OSMF, and clinical TNM staging were the quantitative variables.

## Results

Demographic data

A total of 86 patients, irrespective of age and gender, were included in the study. The demographic details of all the patients included in the study have been specified in Table 3. 

**Table 3:** Demographic data OSMF: oral submucous fibrosis; OSCC: oral squamous cell carcinoma; IQR: interquartile range; OSCC+OSMF: OSCC transformed from OSMF; OSCC-OSMF: OSCC not transformed from OSMF

Parameters	OSMF (n=30)	OSCC+OSMF (n=30)	OSCC-OSMF (n=26)
Age			
Mean age (±SD)	33.67 (±10.14)	45.17 (±9.17)	52.85 (±8.47)
Median age (IQR)	30 (27-39) years	44 (38-52) years	(30-80) years
Age category			
<40 years	23 (76.67%)	9 (30%)	3 (11.53%)
≥40 years	7 (23.33%)	21 (70%)	23 (88.46%)
Gender			
Male	27 (90%)	28 (93.33%)	15 (57.69%)
Female	3 (10%)	2 (6.67%)	11 (42.31%)
Type of habit			
Tobacco	30 (100%)	24 (80%)	16 (61.53%)
Tobacco and alcohol	0 (0%)	6 (20%)	2 (7.70%)
None	0 (0%)	0 (0%)	8 (30.76%)
Habit frequency (pieces/day)			
<10	22 (73.33%)	17 (56.66%)	8 (30.76%)
>10	8 (26.67%)	13 (43.33%)	10 (38.46%)
Habit duration (in years)			
Mean duration (±SD)	6.4 (±4.03)	13.69 (±5.13)	18.7 (±4.2)
Median duration (IQR)	6.5 (2-10)	14 (10-15)	19
Range	1-15	5-25	11-40

Clinicopathological features in OSMF

Mouth opening in OSMF ranged from 10 to 37 mm with a median of 26.5 (23-30) mm and a mean range of 25.53±6.86 mm. Accordingly, two (6.67%) patients were in Stage I, 12 (40%) patients were in Stage II, 11 (36.67%) patients were in Stage III, three (10%) patients were in Stage IVa, and two (6.67%) patients were in Stage IVb. Restricted tongue protrusion was seen in 26 (86.67%) patients, while burning sensation was present in 18 (60%) patients. In nine (30%) patients, there was presence of fibrotic bands in the unilateral buccal mucosa, and in 14 (46.67%) patients, there were fibrotic bands in the bilateral buccal mucosa, whereas in seven (23.33%) patients, fibrotic bands were present in both the bilateral buccal mucosa and labial mucosa. In 25 (83.33%) patients, there was presence of a white patch in the oral mucosa, three (10%) patients had blanching in the unilateral buccal mucosa, 15 (50%) patients had blanching in the bilateral buccal mucosa, and four (13.33%) patients had blanching in both the bilateral buccal mucosa and labial mucosa. The uvula was shrunken in 15 (50%) patients and deviated to one side in two (6.67%) patients.

**Figure 1:**
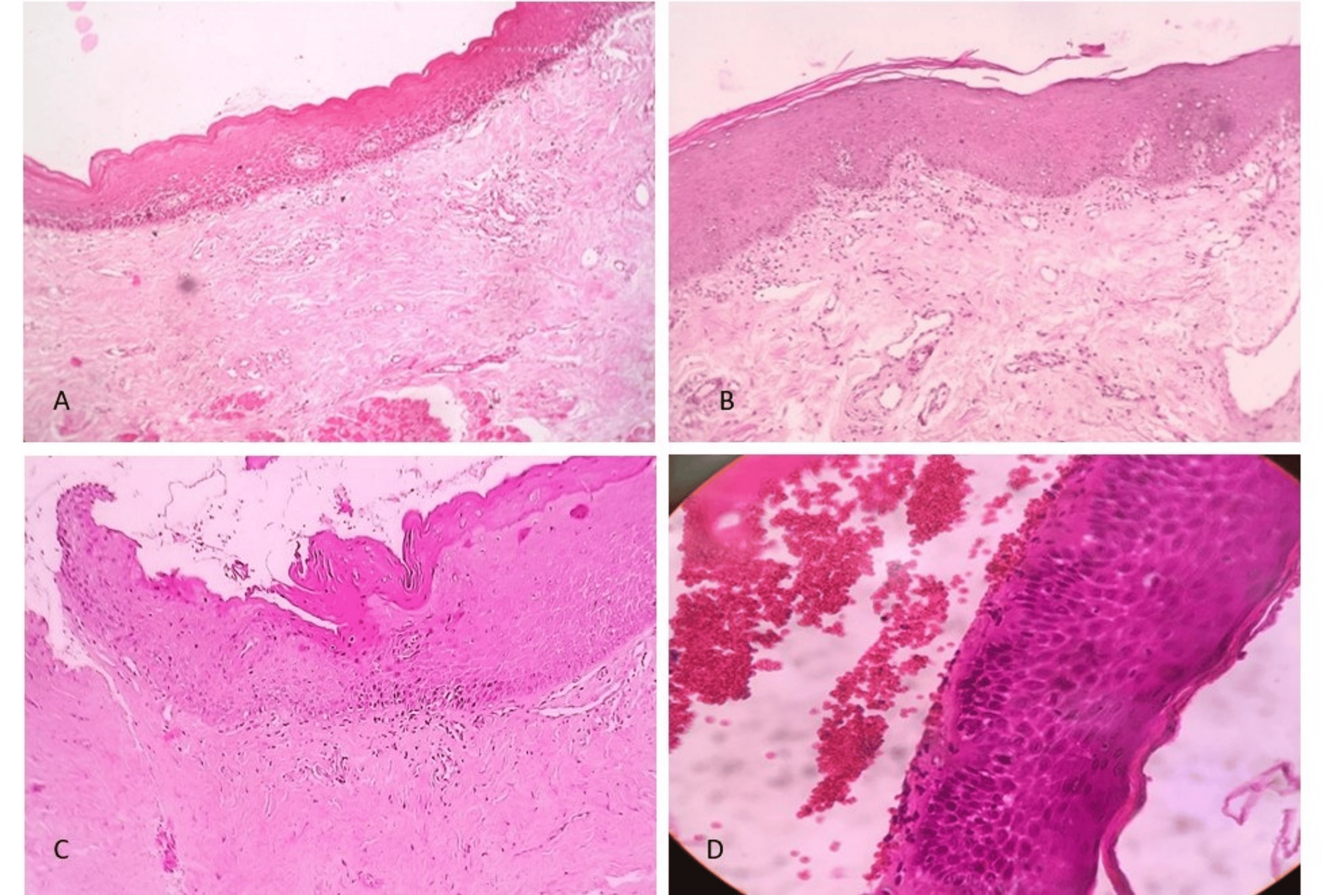
(A) Atrophic epithelium in OSMF (4× magnification). (B) Juxtaepithelial inflammatory cells (4× magnification). (C) Loss of rete ridges and juxtaepithelial inflammatory cells (4× magnification). (D) Juxtaepithelial hyalinisation (10× magnification) OSMF: oral submucous fibrosis

The epithelium was found to be atrophic in all 30 (100%) patients, juxtaepithelial hyalinisation was evident in 10 (33.33%), and juxtaepithelial inflammatory cell infiltration was present in 21 (70%). A mild degree of dysplastic changes was noticed in four (13.33%) patients. Presence of atrophic epithelium, juxtaepithelial inflammatory cells, and juxtaepithelial hyalinisation has been illustrated in Figure 1.

Clinicopathological features in OSCC+OSMF

In the oral cavity, OSCC was diagnosed at a single site in 16 (53.33%) patients, at two different sites in eight (26.66%) patients, and in three different sites in six (20%) patients. Mouth opening ranged from 1 to 28 mm with a median of 15.5 (10-25) mm and a mean range of 16.53±7.35 mm. In nine (30%) patients, fibrotic bands were present in the unilateral buccal mucosa, in seven (23.33%) patients, there were palpable fibrotic bands in the bilateral buccal mucosa, in two (6.67%) patients, there were fibrotic bands in the unilateral buccal mucosa and labial mucosa, and in three (10%) patients, fibrotic bands were present in both the bilateral buccal mucosa and labial mucosa. Five (16.67%) patients were in Stage I, two (6.67%) patients were in Stage II, 12 (40%) patients were in Stage III, and 11 (36.67%) patients were present in Stage IV of TNM staging. On histopathological grading, 18 (60%) patients were diagnosed with well-differentiated OSCC, 11 (36.67%) patients were diagnosed with moderately differentiated OSCC, and one (3.33%) patient was diagnosed with poorly differentiated OSCC.

Clinicopathological features in OSCC-OSMF

The buccal mucosa was the predominant site (50%), followed by the gingivobuccal sulcus (30.77%), lip, tongue, retromolar area, and alveolar ridge. Among 26 specimens, four (15.38%), two (7.69%), 11 (42.31%), and nine (34.62%) were under the clinical TNM staging of Stage I, Stage II, Stage III, and Stage IV, respectively. On histopathological grading, 12 (46.15%) patients were diagnosed with well-differentiated OSCC, 13 (50%) patients were diagnosed with moderately differentiated OSCC, and one (3.58%) patient was diagnosed with poorly differentiated OSCC.

OCT-4 and NANOG immunoexpression

In OSMF, 29 (96.66%) patients showed positive OCT-4 immunoexpression, while in OSCC+OSMF, 28 (93.33%) patients showed positive OCT-4 immunoexpression, and in OSCC-OSMF, 11 (42.30%) patients showed positive immunoexpression of OCT-4. Similarly, in OSMF, 28 (93.33%) patients showed positive NANOG immunoexpression, while in OSCC+OSMF, 25 (83.33%) patients showed positive NANOG immunoexpression, and in OSCC-OSMF, 10 (38.46%) patients showed positive immunoexpression of NANOG. Details of OCT-4 and NANOG immunoexpression in OSMF, OSMF+OSCC, and OSMF-OSCC have been described in Table 4.

**Table 4:** Details of OCT-4 and NANOG immunoexpression OSMF: oral submucous fibrosis; OSCC: oral squamous cell carcinoma; OSCC+OSMF: OSCC transformed from OSMF; OSCC-OSMF: OSCC not transformed from OSMF

Features	OCT-4			NANOG		
	OSMF; n=30 (%)	OSCC+OSMF; n=30 (%)	OSCC-OSMF; n=26 (%)	OSMF; n=30 (%)	OSCC+OSMF; n=30 (%)	OSCC-OSMF; n=26 (%)
No. of positive cases	29 (96.66)	28 (93.33)	11 (42.30)	28 (93.33)	25 (83.33)	10 (38.46)
Tissue localisation						
Epithelium	16 (53.33)	1 (3.33)	1 (3.84)	4 (13.33)	0 (0)	1 (3.84)
Connective tissue	2 (6.67)	3 (10)	4 (15.38)	3 (10)	3 (10)	5 (19.23)
Epithelium+connective tissue	11 (36.67)	24 (80)	6 (23.07)	21 (70)	22 (73.33)	4 (15.38)
N/A	1 (3.33)	2 (6.67)	15 (57.69)	2 (6.67)	5 (16.67)	16 (61.53)
Epithelial layer localisation						
Basal layer	8 (26.67)	-	-	0 (0)	-	-
Parabasal layer	0 (0)	-	-	0 (0)	-	-
Basal+parabasal layer	19 (63.33)	-	-	25 (83.33)	-	-
N/A	3 (10)	-	-	5 (16.67)	-	-
Distribution in tumour islands						
Periphery of islands	-	18 (60)	4 (15.38)	-	14 (46.67)	5 (19.23)
Complete island	-	8 (26.67)	7 (26.92)	-	10 (33.33)	5 (19.23)
N/A	-	4 (13.33)	15 (57.69)	-	6 (20)	16 (61.53)
Cellular localisation						
Nucleus	11 (36.67)	1 (3.33)	3 (11.53)	9 (30)	2 (6.67)	3 (11.53)
Cytoplasm	6 (20)	2 (6.67)	3 (11.53)	5 (16.67)	6 (20)	4 (15.38)
Nucleus+cytoplasm	12 (40)	25 (83.33)	5 (19.23)	14 (46.67)	17 (56.67)	3 (11.53)
N/A	1 (3.33)	2 (6.67)	15 (57.69)	2 (6.67)	5 (16.67)	16 (61.53)
Qualitative expression						
0	1 (3.33)	2 (6.67)	15 (57.69)	2 (6.67)	5 (16.67)	16 (61.53)
1	4 (13.33)	5 (16.67)	2 (7.69)	2 (6.67)	3 (10)	1 (3.84)
2	7 (23.33)	19 (63.33)	2 (7.69)	10 (36.67)	14 (46.67)	6 (23.07)
3	18 (60)	4 (13.33)	7 (26.92)	16 (53.33)	8 (26.67)	3 (11.53)
Quantitative expression						
0	1 (3.33)	2 (6.67)	15 (57.69)	2 (6.67)	5 (16.67)	16 (61.53)
1	1 (3.33)	1 (3.33)	2 (7.69)	1 (3.33)	0 (0)	1 (3.84)
2	16 (53.33)	12 (40)	4 (15.38)	11 (36.67)	8 (26.67)	6 (23.07)
3	12 (40)	15 (50)	5 (19.23)	16 (53.33)	17 (56.67)	3 (11.53)
Combinative expression						
0	1 (3.33)	2 (6.67)	15 (57.69)	2 (6.67)	5 (16.67)	16 (61.53)
1	1 (3.33)	1 (3.33)	0 (0)	0 (0)	0 (0)	0 (0)
2	3 (10)	4 (13.33)	0 (0)	1 (3.33)	2 (6.67)	0 (0)
3	0 (0)	0 (0)	1 (3.84)	2 (6.67)	1 (3.33)	0 (0)
4	1 (3.33)	8 (26.67)	0 (0)	2 (6.67)	5 (16.67)	1 (3.84)
6	18 (60)	11 (36.67)	5 (19.23)	16 (53.33)	10 (33.33)	6 (23.07)
9	6 (20)	4 (13.33)	5 (19.23)	7 (23.33)	7 (23.33)	3 (11.53)

**Figure 2:**
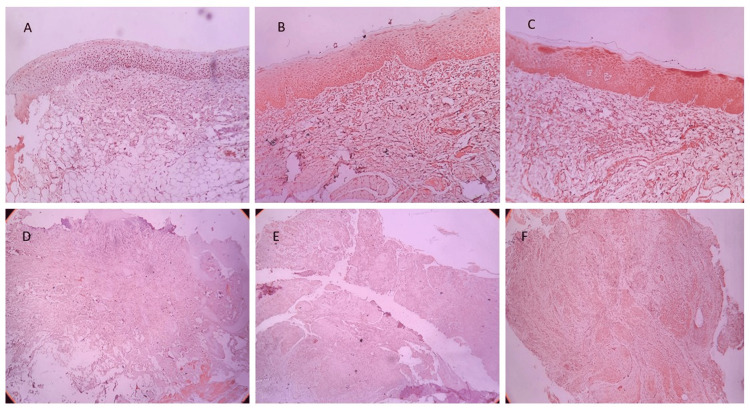
Immunohistochemical staining of OCT-4 under 4× magnification: (A) Weak OCT-4 immunoexpression in OSMF. (B) Moderate OCT-4 immunoexpression in OSMF. (C) Strong OCT-4 immunoexpression in OSMF. (D) Weak OCT-4 immunoexpression in OSCC+OSMF. (E) Moderate OCT-4 immunoexpression in OSCC+OSMF. (F) Strong OCT-4 immunoexpression in OSCC+OSMF OSMF: oral submucous fibrosis; OSCC: oral squamous cell carcinoma; OSCC+OSMF: OSCC transformed from OSMF

**Figure 3:**
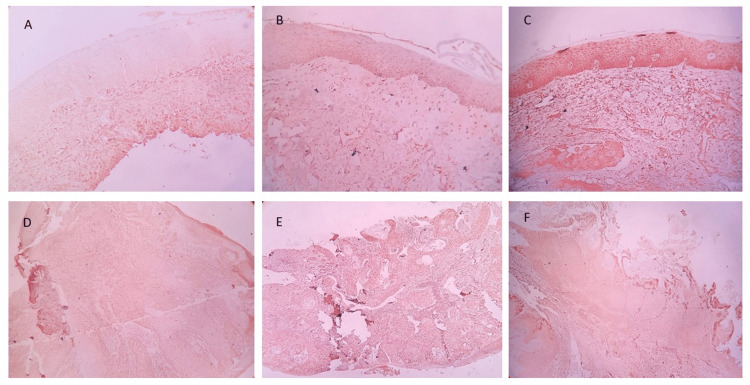
Immunohistochemical staining of NANOG under 4× magnification: (A) Weak NANOG immunoexpression in OSMF. (B) Moderate NANOG immunoexpression in OSMF. (C) Strong NANOG immunoexpression in OSMF. (D) Weak NANOG immunoexpression in OSCC+OSMF. (E) Moderate NANOG immunoexpression in OSCC+OSMF. (F) Strong NANOG immunoexpression in OSCC+OSMF OSMF: oral submucous fibrosis; OSCC: oral squamous cell carcinoma; OSCC+OSMF: OSCC transformed from OSMF

Weak, moderate, and strong OCT-4 and NANOG immunoexpression in OSMF and OSMF+OSCC have been illustrated in Figure 2 and Figure 3, respectively.

Comparison of OCT-4 immunoexpression in OSMF, OSCC+OSMF, and OSCC-OSMF

Comparison of the Number of Positive Cases Among the Three Groups

**Figure 4:**
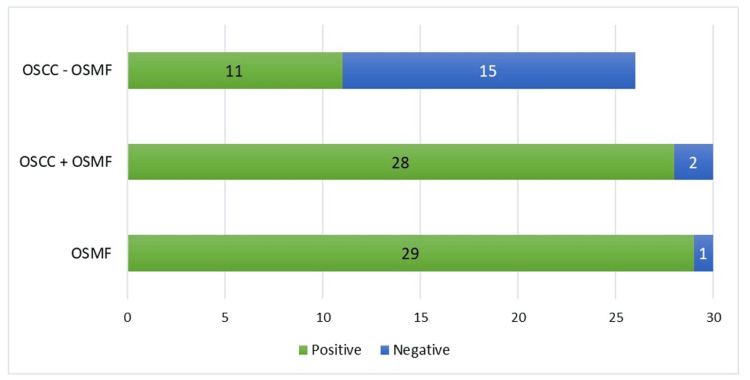
Comparison of OCT-4 immunoexpression among the three groups OSMF: oral submucous fibrosis; OSCC: oral squamous cell carcinoma; OSCC+OSMF: OSCC transformed from OSMF; OSCC-OSMF: OSCC not transformed from OSMF

On comparing the number of positive cases between OSMF, OSCC+OSMF, and OSCC-OSMF patients, the data was found to be statistically significant. The unadjusted odds ratio was 5.23 (95% CI: 0.10-2.67; p<0.001) indicating that OSCC+OSMF patients had a substantially higher likelihood of positive OCT-4 immunoexpression as compared to their OSCC-OSMF counterparts, as illustrated in Table 5 and Figure 4. 

**Table 5:** Comparison of OCT-4 immunoexpression among the three groups Odds ratio between OSCC+OSMF and OSCC-OSMF: 5.23; 95% CI: 0.10-2.67; p<0.001 OSMF: oral submucous fibrosis; OSCC: oral squamous cell carcinoma; OSCC+OSMF: OSCC transformed from OSMF; OSCC-OSMF: OSCC not transformed from OSMF

Conditions	OCT-4 immunoexpression		Fisher's exact test
	Positive	Negative	
OSMF	29 (96.67%)	1 (3.33%)	<0.001
OSCC+OSMF	28 (93.33%)	2 (6.67%)	
OSCC-OSMF	11 (42.31%)	15 (57.69%)	

Comparison of Staining Intensity Among the Three Groups

**Figure 5:**
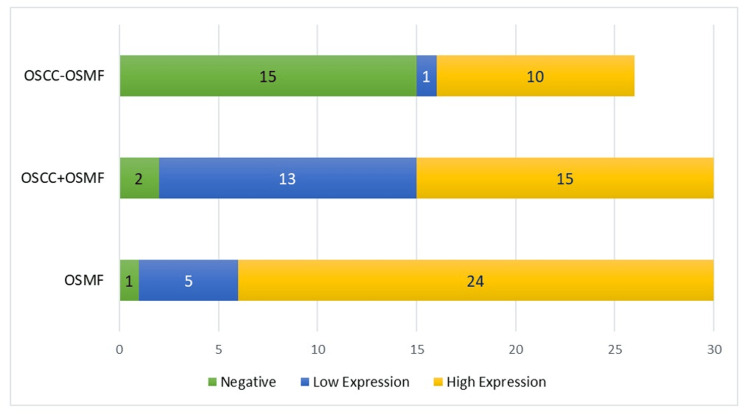
Comparison of OCT-4 staining intensity among the three groups OSMF: oral submucous fibrosis; OSCC: oral squamous cell carcinoma; OSCC+OSMF: OSCC transformed from OSMF; OSCC-OSMF: OSCC not transformed from OSMF

On comparing staining intensity between OSMF, OSCC+OSMF, and OSCC-OSMF patients, the data was found to be statistically significant (p<0.001). The unadjusted odds ratio was 1.08 (95% CI: 0.55-1.83; p<0.001) indicating that OSCC+OSMF patients had a substantially higher OCT-4 staining intensity as compared to their OSCC-OSMF counterparts, as illustrated in Table 6 and Figure 5. 

**Table 6:** Comparison of OCT-4 staining intensity among the three groups Odds ratio between OSCC+OSMF and OSCC-OSMF: 1.08; 95% CI: 0.55-1.83; p<0.001 OSMF: oral submucous fibrosis; OSCC: oral squamous cell carcinoma; OSCC+OSMF: OSCC transformed from OSMF; OSCC-OSMF: OSCC not transformed from OSMF

Conditions	OCT-4 immunoexpression			Fisher's exact test
	Negative	Low expression	High expression	
OSMF	1 (3.33%)	5 (16.67%)	24 (80%)	<0.001
OSCC+OSMF	2 (6.67%)	13 (43.33%)	15 (50%)	
OSCC-OSMF	15 (57.69%)	1 (3.85%)	10 (38.46%)	

Comparison of NANOG immunoexpression in OSMF, OSCC+OSMF, and OSCC-OSMF

Comparison of the Number of Positive Cases Among the Three Groups

**Figure 6:**
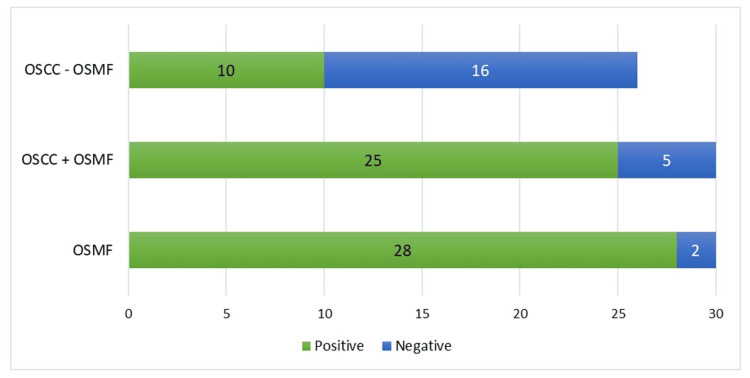
Comparison of NANOG immunoexpression among the three groups OSMF: oral submucous fibrosis; OSCC: oral squamous cell carcinoma; OSCC+OSMF: OSCC transformed from OSMF; OSCC-OSMF: OSCC not transformed from OSMF

On comparing the number of positive cases between OSMF, OSCC+OSMF, and OSCC-OSMF patients, the data was found to be statistically significant. The unadjusted odds ratio was 12.5 (95% CI: 0.36-4.33; p<0.001) indicating that OSCC+OSMF patients had a substantially higher likelihood of positive NANOG immunoexpression as compared to their OSCC- OSMF counterparts, as illustrated in Table 7 and Figure 6. 

**Table 7:** Comparison of NANOG immunoexpression among the three groups Odds ratio between OSCC+OSMF and OSCC-OSMF: 12.5; 95% CI: 0.36-4.33; p<0.001 OSMF: oral submucous fibrosis; OSCC: oral squamous cell carcinoma; OSCC+OSMF: OSCC transformed from OSMF; OSCC-OSMF: OSCC not transformed from OSMF

Conditions	NANOG expression		Fisher’s exact test
	Positive	Negative	
OSMF	28 (93.33%)	2 (6.67%)	<0.001
OSCC+OSMF	25 (83.33%)	5 (16.67%)	
OSCC-OSMF	10 (38.46%)	16 (61.54%)	

Comparison of Staining Intensity Among the Three Groups

**Figure 7:**
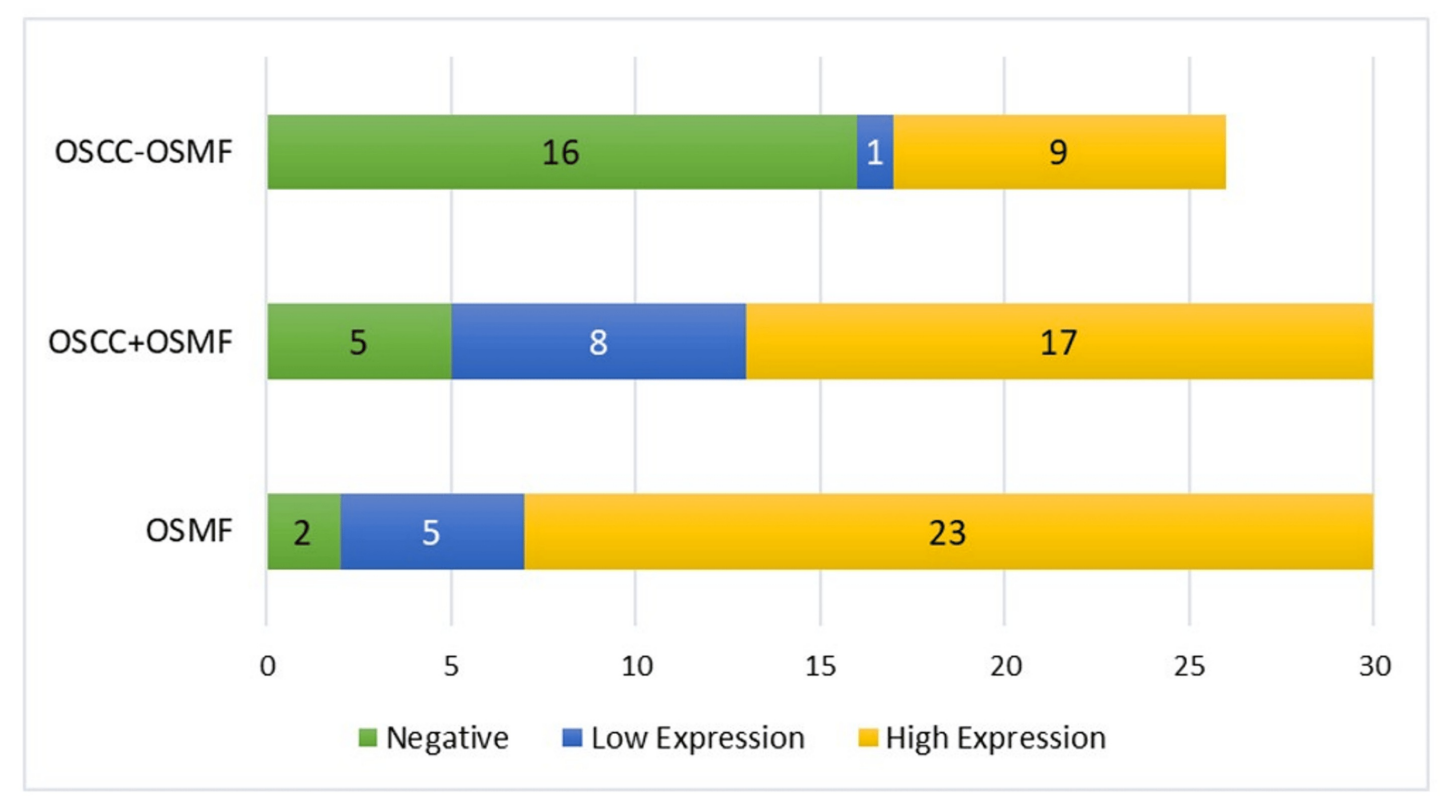
Comparison of NANOG staining intensity among the three groups OSMF: oral submucous fibrosis; OSCC: oral squamous cell carcinoma; OSCC+OSMF: OSCC transformed from OSMF; OSCC-OSMF: OSCC not transformed from OSMF

On comparing staining intensity between OSMF, OSCC+OSMF, and OSCC-OSMF patients, the data was found to be statistically significant (p<0.001). The unadjusted odds ratio was 1.06 (95% CI: 0.53-1.88; p<0.001) indicating that OSCC+OSMF patients had a substantially higher NANOG staining intensity as compared to their OSCC-OSMF counterparts, as illustrated in Table 8 and Figure 7.

**Table 8:** Comparison of NANOG staining intensity among the three groups Odds ratio between OSCC+OSMF and OSCC-OSMF: 1.06; 95% CI: 0.53-1.88; p<0.001 OSMF: oral submucous fibrosis; OSCC: oral squamous cell carcinoma; OSCC+OSMF: OSCC transformed from OSMF; OSCC-OSMF: OSCC not transformed from OSMF

Conditions	NANOG expression			Fisher's exact test
	Negative	Low expression	High expression	
OSMF	2 (6.67%)	5 (16.67%)	23 (76.67%)	<0.001
OSCC+OSMF	5 (16.67%)	8 (26.67%)	17 (56.67%)	
OSCC-OSMF	16 (61.54%)	1 (3.85%)	9 (34.62%)	

## Discussion

OSCC+OSMF is believed to constitute a distinct clinicopathological entity attributed to the differences in clinical and histopathological features and the unique areca nut-induced carcinogenesis mechanisms. In addition to extrapolating further differences, we investigated the differences in the immunohistochemical expression of OCT-4 and NANOG between these two groups. We then compared the immunohistochemical expression of these two markers in OSCC+OSMF and OSCC-OSMF to predict the role of OCT-4 and NANOG in the malignant transformation of OSMF. Descriptive analysis of the study population showed that the mean age of presentation in OSMF was 33.67 (±10.14) years and, in the OSCC+OSMF group, it was found to be 45.17 (±9.17) years. This finding is close to the mean age of 48.78 years as reported by a recent meta-analysis [19]. The mean age of OSCC-OSMF was found to be 52.85 which is higher than OSCC+OSMF. These findings are well supported by Acharya et al. [20], where the mean age of OSCC+OSMF and OSCC-OSMF were found to be 40.5 and 54 years, respectively. In both OSMF and OSCC+OSMF, males were nine times and 14 times more predominant than females. In OSCC-OSMF, the male-to-female proportion was found to be 15:11. In the OSCC-OSMF group, eight (30.76%) patients did not have any history of tobacco consumption, out of which five (19.23%) were female patients. This high prevalence of OSCC-OSMF may be attributed to etiological factors like trauma and factors other than tobacco in female patients. This supports the fact of the absence of traditional risk factors of oral cancer in females as reported by Papageorge [21].

Fibrosis and hyalinisation in and around minor salivary glands can lead to a reduction in saliva secretion, which can cause a lack of membrane-associated mucin on the tips of microplicae resulting in a reduction of salivary mucous gel secretion locally. Consequently, there occurs less protection of the oral mucous membrane against irritation from spicy and hot food [22]. As OSMF progresses towards malignancy, mouth opening is further impaired which is evident in the present study where the mean mouth opening in the non-transformed group is 25.53±6.86 mm which is reduced to 16.53±7.35 mm in the transformed group. Limited tongue protrusion was seen in the majority (86.67%) of patients having OSMF, while shrunken uvula was a prevalent feature among OSMF patients. Among OSMF patients, 86.67% did not show any dysplastic features, which contradicts the findings of a report on the clinicopathological features of OSMF where mild, moderate, and severe epithelial dysplasia were observed in 46%, 52%, and 2% of cases, respectively [23]. Considering the fact that all the cases of the OSMF group were showing atrophic epithelium, our findings are in accordance with Sanjai who criticised the perpetuation of oral epithelial dysplasia criteria in the atrophic epithelium of OSMF [24]. In the OSMF+OSCC group, 12 (40%) patients were in Stage III, and 11 (36.67%) patients were in Stage IV. Similarly, in the OSCC group, 11 (42.31%) patients were in Stage III, while nine (34.62%) patients were in Stage IV, which highlights that the advanced stage of presentation can be related to a lack of awareness among the Indian population [25]. There is a continuous inquisitiveness among the researchers to understand the mechanism of the malignant transformation of OSMF. In addition to OCT-4 and NANOG, if any markers can prove to have predictive potential for the malignant transformation of OSMF, that would help clinicians in managing the patients in a better way.

The association between CSCs and EMT has been established, and their similarities have been analysed to play a crucial role in cancer metastasis, tumour recurrence, and therapy resistance. Studies suggest that cells undergoing EMT can acquire stem cell-like properties as in breast epithelial cells where induced EMT has shown properties similar to mesenchymal cells like genetic expression, multidirectional differentiation, and migration towards wound sites [12]. EMT induction in human mammary cells has also shown an increased expression of mesenchymal markers like CD44+ and CD24− which accounts for enhanced abilities of cellular invasion, migration, and proliferation [11]. The immunoexpression of OCT-4 and NANOG was significantly less in OSCC-OSMF as compared to OSCC+OSMF, which suggests the significant role of OCT-4 and NANOG in the malignant transformation of OSMF.

Immunoexpression of OCT-4 was seen in both the nucleus and cytoplasm. This staining pattern is due to the presence of two isoforms of OCT-4 such as OCT-4A and OCT-4B which have also been observed in the nucleus and cytoplasm of prostate and cervical cancers, respectively [26]. OCT-4 and NANOG are considered as embryonic marker master regulators due to their major involvement in the reprogramming of somatic cells into embryonic stem cell-like cells. Upregulation of OCT-4 and NANOG immunoexpression is observed in areca nut-induced carcinogenesis which complies with the findings of our study [27]. Oral cancers among habitual areca nut chewers demonstrate an aggressive phenotype, chemoradioresistance, and a much lower five-year survival rate than those without areca habits [28]. OCT-4 and SOX2 are upregulated due to the downregulation of miR-145 by arecoline. As miR-145 is a positive regulator of E-cadherin and a negative regulator of Snail (SNAI1) and Slug (SNAI2), EMT occurs. The exposed epithelial cells thus acquire increased chemoresistance, augmented migration, increased invasiveness, and anchorage-independent growth. Furthermore, SOX2 and OCT-4 expression is inversely related to miR-145 expression in the tissues of individuals with areca quid-induced OSCC [29]. Since miR-145 downregulates c-MYC, the suppression of miR-145 drives the upregulation of c-MYC [30].

While comparing OCT-4 intensity and the number of positive cases in three groups such as OSMF, OSCC+OSMF, and OSCC-OSMF, we identified a progressive decrease in intensity and the number of positive cases from OSMF to OSCC+OSMF to OSCC-OSMF. The odds of OCT-4 expression in OSCC was 5% lower (0.052381) as compared to that of OSCC+OSMF. Similarly, NANOG expression was also found to decrease in intensity, and the number of positive cases also decreased from OSMF to OSCC+OSMF to OSCC-OSMF. The odds of low expression of NANOG was 12% less (0.125) in OSCC+OSMF as compared to OSCC-OSMF. Studies suggest that high levels of OCT-4 and NANOG are associated with the early stage of disease and better prognosis [31]. Corroborating these findings, along with our findings, we may suggest that both these markers have a protective effect on OSCC+OSMF.

CSC and EMT are two important molecular pathways of tumour progression in OSCC. While CSC is critical for initiation, EMT is important for tumour progression. Both mechanisms are mutually inclusive. OCT-4 and NANOG regulate the stemness properties of tumour and promote metastasis in lung cancer [30]. The expression of OCT-4 has further been shown in human breast cancer stem-like cells, implicating its involvement in tumorigenesis and self-renewal by activating its downstream target genes, such as NANOG and SOX2. NANOG, a downstream target of OCT-4, contributes to cell fate determination of the pluripotent inner cell mass during embryonic development [32]. It promotes CSC characteristics in prostate cancer and regulates the self-renewal of CSC in human hepatocellular carcinoma (HCC) [33]. Several lines of evidence have suggested that the expression of OCT-4 and NANOG is closely related to tumorigenesis, distant recurrence, and tumour metastasis after treatment [30,34]. Higher expression of CSC markers like OCT-4 and NANOG in OSMF and OSCC+OSMF is well supported by the previous studies which have observed the increased expression of other CSCs in OSMF and the malignant transformation of OSMF [35]. Increased expression of these two molecules in OSCC+OSMF as compared to OSCC-OSMF as found in this study may suggest the increased activity of OCT-4- and NANOG-mediated stem cell-induced EMT in the malignant transformation of OSMF. While the present study focuses on the role of CSC markers OCT-4 and NANOG in the malignant transformation of OSMF, other CSC markers like survivin [36] and CD105 [37] have also been identified in OSMF and OSCC+OSMF cases, and further studies on these markers could provide a more comprehensive understanding of the molecular mechanisms underlying the progression of OSMF to OSCC.

A major limitation of the present study investigating OCT-4- and NANOG-mediated tumour progression in OSCC+OSMF is the small sample size. Long-term follow-up of these patients would provide valuable insights into the prognostic influence of these markers on OSCC survival outcomes.

## Conclusions

OSCC, like many solid tumours, contains a heterogeneous population of cancer cells. The side population of cancer cells is reportedly enriched with CSCs; however, the functional role and clinical relevance of CSC markers in the cell population of OSCC are yet to be elucidated. The study highlights the potential role of OCT-4 and NANOG as key molecular indicators in the malignant transformation of OSMF. Their elevated immunoexpression in OSMF and OSCC+OSMF underscores their involvement in CSC regulation and EMT mechanisms that facilitate tumour initiation and progression. The findings suggest that OCT-4 and NANOG are not merely markers of pluripotency but active contributors to the pathogenesis of OSCC. Assessing their expression patterns may enhance the early detection of malignant potential in OSMF and assist in identifying patients at greater risk of developing OSCC.
